# Orf virus infection of the hand in a Scottish sheep farmer. A case report to increase awareness to avoid misdiagnosis

**DOI:** 10.1080/23320885.2021.2016057

**Published:** 2021-12-13

**Authors:** Yasmeen Khan, Jordon Currie, Clare Miller, David Lawrie

**Affiliations:** Department of Trauma and Orthopaedics, NHS Grampian, Aberdeen Royal Infirmary, Aberdeen, Scotland

**Keywords:** Orf infection, hand, trauma

## Abstract

Awareness of infections which are transmitted between animals and humans have been given prominence following the (COVID-19) pandemic. The Orf infection in humans is rare. Recognition of Orf lesions avoids misdiagnosing and incorrect treatment. We present a case of a Scottish-farmer with pain and discomfort from a lesion on her finger.

## Introduction

Awareness of infections which are transmitted between animals and humans have been given prominence due to the devastation caused by the novel severe acute respiratory syndrome coronavirus 2 (SARS-CoV-2) or more commonly known as the coronavirus. The Orf virus infection is a zoonotic parapox virus carried by sheep and goats [1]. The infected animal presents with scabs around the mouth, this has resulted in the infection commonly known as scabby mouth disease or sore mouth disease [2]. Agricultural workers, vets, sheep shearers, butchers and other humans who are in contact with infected sheep are susceptible to the infection. In humans, the hand is the most common site of manifestation resulting in skin lesions [3]. The virus is a parapoxvirus containing DNA and is transmitted directly thorough open wounds [4]. The incubation period varies up to two weeks post exposure. The patient can have local signs on the hand but also present with extra skeletal manifestation such as fever, malaise and lymphadenopathy. If the patient is immunocompromised the severity of infection can be significant causing long term morbidity [5]. The skin lesions have specific features which suggest an Orf virus infection. This is the case report of the orf virus infection in a Scottish sheep farmer from Aberdeenshire.

## Case presentation

A 47-year-old female, Scottish Sheep farmer presented to Aberdeen Royal Infirmary Major Trauma unit with pain and discomfort from a lesion on her non dominant left ring finger distal phalanx. She accidently stabbed her hand when she was shearing a sheep this resulted in a laceration to her left ring finger. She presented 2 weeks following the incident with a soft well circumscribed mass on the ulnar aspect of her distal phalanx bordering the nail plate. The patient has a past medical history of protein c deficiency for which she requires lifelong warfarin. She is a non-smoker, works full time as a farmer and is physically fit able to perform all activities of daily living. She was not aware that any of her sheep were infected with the Orf virus infection and had not seen evidence of lesions around the mouths of her sheep.

**Figure 1.**
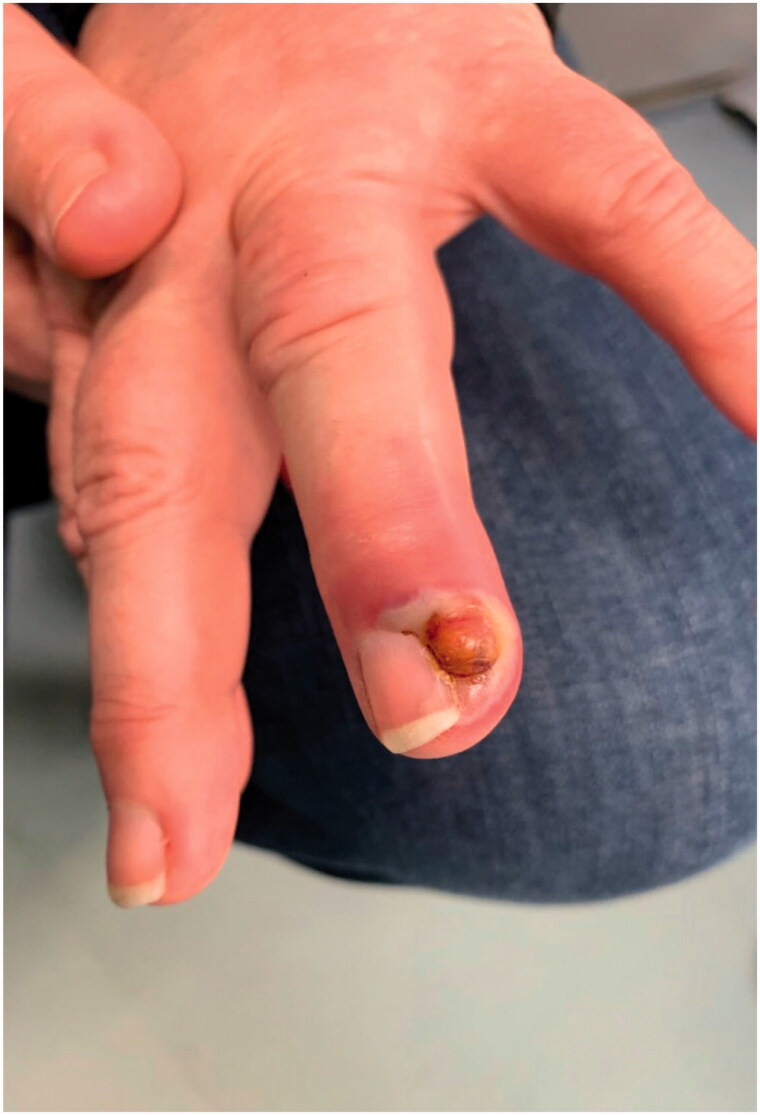
Clinical image of the lesion present on the left ring finger.

**Figure 2.**
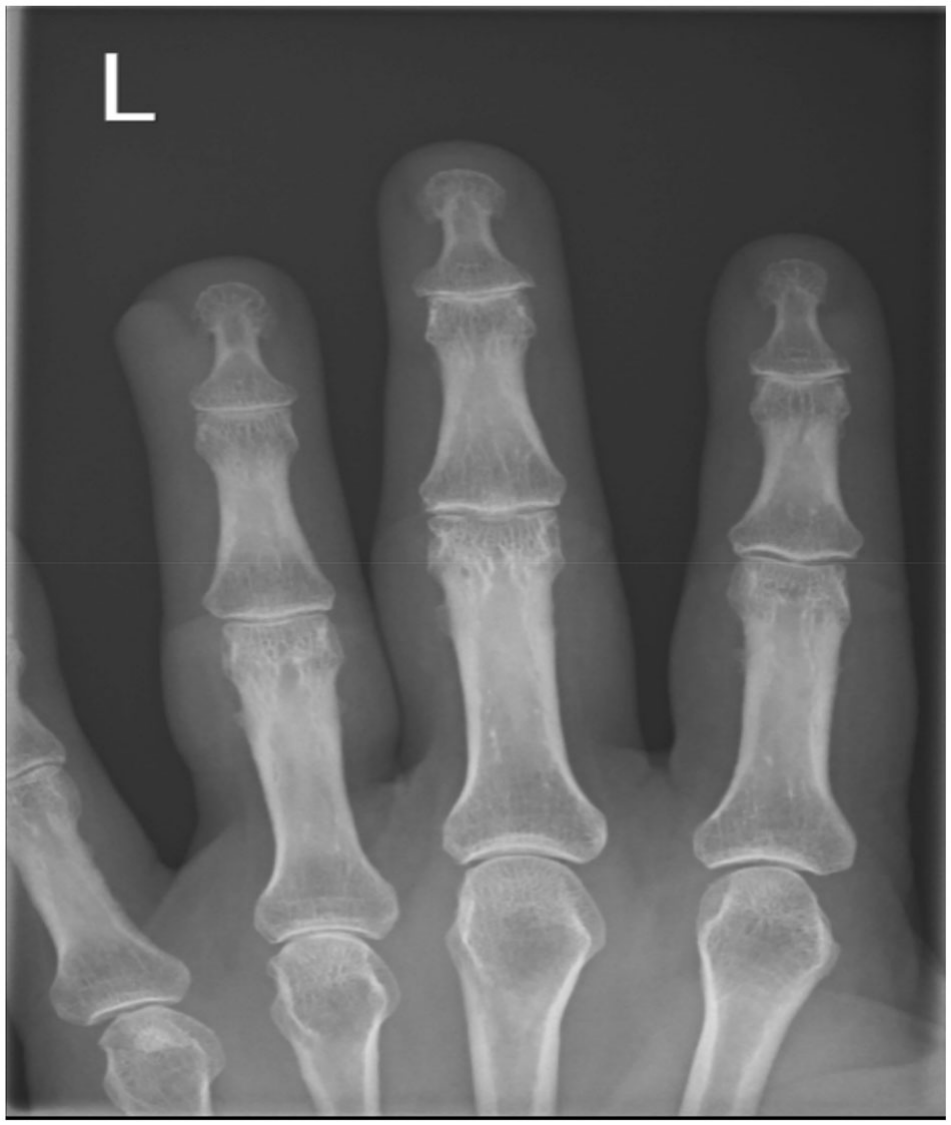
Radiographs of the left hand ring finger – PA view of the Left Index, Middle and Ring Fingers no obvious osseous pathology, left ring finger – increase density over the distal ulnar aspect of the left ring finger.

**Figure 3.**
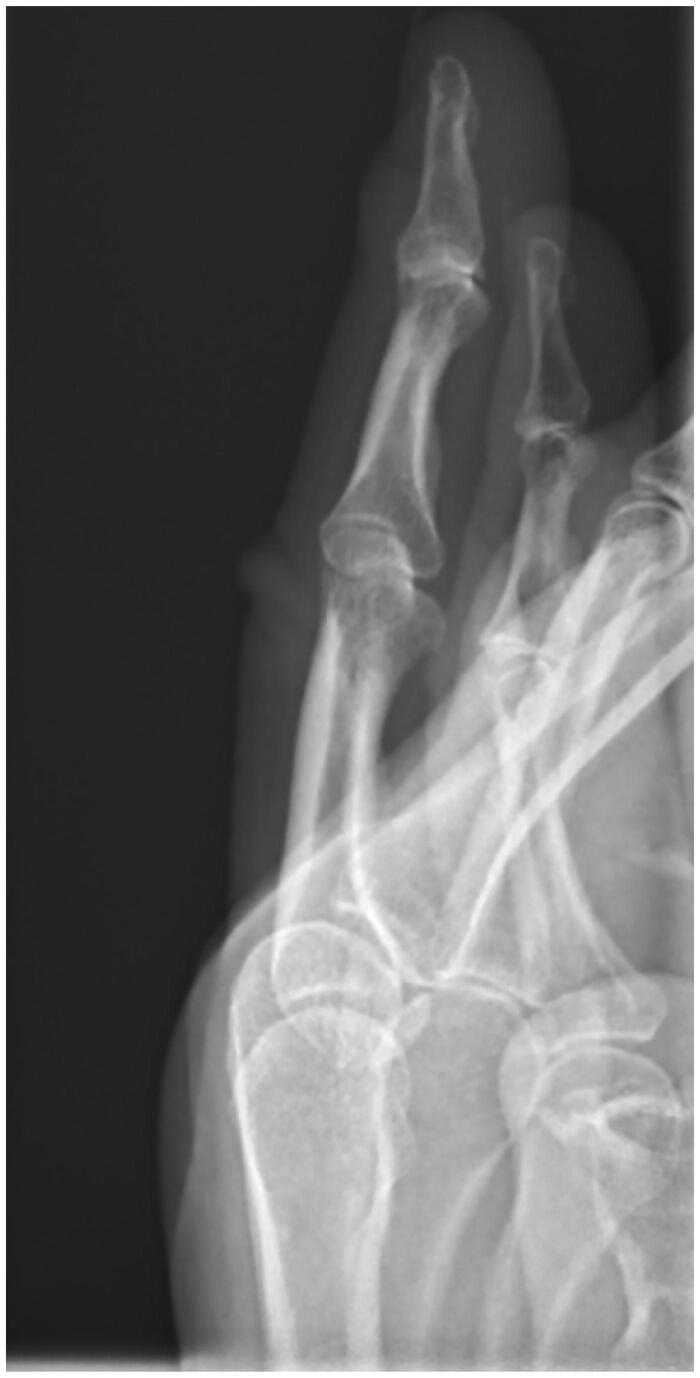
Lateral radiograph of the left ring finger.

Aberdeen Royal Infirmary run a dedicated tertiary hand service. The on-call orthopaedic hand registrar reviewed the patient. After taking a full medical history a through clinical examination was performed. The lesion presented on the left ring finger distal ulnar aspect bordering the nail a 4 mm by 4 mm discrete brown hemispherical lesion was noted (Figure 1). The surrounding skin was slightly erythematous. The lesion was not warm, nor tender on palpation. The patient did not present any Kanaval’s signs to suggest pyogenic tenosynovitis or evidence of dorsal compartment infection. No fluctuance was noted in the lesion. The patient had a full range of movement in her left ring finger. The distal neurovascular status of the finger was intact. The patient had seen a clear fluid exudate discharge a day before she presented. The lesion was dry on presentation. The patient was prescribed oral antibiotics by her general practitioner which commenced a week before presenting to the hand unit. The antibiotics did not resolve the lesion. Radiographs taken did not show any evidence of foreign body within the distal aspect of the left ring finger (Figures 2 and 3).

The differential diagnosis included over granulation following trauma to the hand, pyogenic granuloma or a felon. Her observations and inflammatory markers were normal, this further ruled out infection. The patient presented to the hand trauma clinic 2 weeks after presentation to the hand on call service. Her hand was re-examined the lesion showed evidence of regression. The senior author recognised the lesion. The Orf virus infection diagnosis in a non-immunocompromised patient is self- limiting. The lesion eventually resolved in 10–12 weeks without any scars.

## Discussion

Trauma and orthopaedic surgeons, plastic surgeons, accident and emergency teams and general practitioners who are involved in the primary care of patients should be familiar with the Orf virus infection in humans. Lesions are often misdiagnosed. The rapidly growing mass can be mistaken as a malignant tumour. There have been reports of patients undergoing amputation due to a misdiagnosis, which is devastating as the Orf virus infection is self-limiting with full resolution of the lesions [6].

The origin of the word Orf is derived from hrufa which is Nordic in origin, meaning boil or scab [5]. The Orf virus infection is well recognised in Western Asia, North Africa, Western Africa and Eastern Africa. Migration, free travel to countries around the world has resulted in the spread of the Orf virus infection. This has resulted in an outbreak of Orf virus infection in humans living in a farm in Europe. There have been reported cases in Canada and Israel [1,7].

The clinical presentation can be variable depending on the health of the host. The incubation period has been reported to be approximately 2 weeks from transmission [1,4,5]. Healthy patients usually present with a single skin lesion often found on the hand. The lesion progresses through a cycle of stages form presentation to resolution at approximately 8 weeks [8]. There are 6 stages of the lesion the initial stage begins with a maculo-papular stage with erythematous papule, the lesion is red/brown in colour and has a halo this is called a target lesion and represents the acute phase. The next stage is when the lesion weeps a clear fluid, this is called the active phase and then progresses to become dry known as the regenerative stage. This results in a papilloma-like lesion referred to as the papillomatous stage. The lesion forms a dry crust which then spontaneously resolves with minimal scarring [9].

An immunocompromised patient can suffer from severe morbidity from the virus resulting in fever, malaise and lymphadenopathy. There is T cell dysfunction resulting in giant Orf or multiple lesions. They may further manifest skin lesion including Bullous pemphigoid, erythema multiforme. They have a protracted course of the illness or failure of spontaneous resolution of the lesions [5].

The Orf hand lesion is rare, due to the lack of familiarity and recognition of the lesion there have been multiple reports of misdiagnosis with catastrophic consequences [5,6]. The Orf virus is just one of a number of zoonotic poxvirus infections known to affect farmers and animal handlers. Other animals such as cattle, dear and wildlife species such as seals also can cause infection.

The lesion can be misdiagnosed as an acute bacterial infection as visually (Figure 1) in the acute phase it resembles a paronychia or a felon. Oral antibiotics were initially provided to this patient due to a lack of awareness. Patients who progress to an incision and drainage are at risk of secondary bacterial infections [10]. Further differential diagnosis include herpes simplex infection, mycobacterium marinum (fish tank granuloma), pyogenic granuloma and keratoacanthoma. Potentially lethal infections include tularemia (from rabbits) and cutaneous anthrax (from sheep and goats) which also resemble Orf lesions [4].

The Orf virus infection is self-limiting resolving in 10–12 weeks. In healthy individuals even though the lesion is unsightly non operative management is recommended. Advice to keep the lesion clean using aseptic to avoid secondary bacterial infection is recommended to avoid secondary bacterial infections [10]. Topical imidazoquinalines (Imiquimod) have been shown to stimulate the local pro-inflammatory cytokines resulting in a shorter recovery [11]. The immunocompromised patient is at risk of a long recovery and a risk of persistent lesions. Topical Imiquimod has been more effective than antiviral ointments and is recommended for immunocompromised patients. Gaint lesions which are resistant to medical treatments require surgical excision with removal of normal surrounding skin as there have been reports of recurrence at the skin resection margins [5].

Preventative measure and increased awareness is required to professionals working with livestock. The use of gloves helps to prevent direct and indirect exposure. Vaccinations of flocks aims to control the Orf virus infection but animals regardless of being vaccinated can still infect workers through direct contamination [12].

## Conclusion

The Orf virus infection is rare. The importance of spreading awareness of the resultant hand lesions is vital to the correct management of patients in order to prevent misdiagnosis.
